# A Tactile Sensor Using Piezoresistive Beams for Detection of the Coefficient of Static Friction

**DOI:** 10.3390/s16050718

**Published:** 2016-05-18

**Authors:** Taiyu Okatani, Hidetoshi Takahashi, Kentaro Noda, Tomoyuki Takahata, Kiyoshi Matsumoto, Isao Shimoyama

**Affiliations:** 1 Department of Mechano-Informatics, Graduate School of Information Science and Technology, The University of Tokyo, 7-3-1 Hongo, Bunkyo-ku, Tokyo 113-8656, Japan; okatani@leopard.t.u-tokyo.ac.jp (T.O.); takahashi@leopard.t.u-tokyo.ac.jp (H.T.); noda@leopard.t.u-tokyo.ac.jp (K.N.); takahata@leopard.t.u-tokyo.ac.jp (T.T.); 2 Department of Mechanical Engineering, Faculty of Science and Engineering, Toyo University, 2100 Kujirai, Kawagoe-shi, Saitama 350-8585, Japan; matsumoto064@toyo.jp

**Keywords:** tactile sensor, piezoresistive, coefficient of static friction, robotics

## Abstract

This paper reports on a tactile sensor using piezoresistive beams for detection of the coefficient of static friction merely by pressing the sensor against an object. The sensor chip is composed of three pairs of piezoresistive beams arranged in parallel and embedded in an elastomer; this sensor is able to measure the vertical and lateral strains of the elastomer. The coefficient of static friction is estimated from the ratio of the fractional resistance changes corresponding to the sensing elements of vertical and lateral strains when the sensor is in contact with an object surface. We applied a normal force on the sensor surface through objects with coefficients of static friction ranging from 0.2 to 1.1. The fractional resistance changes corresponding to vertical and lateral strains were proportional to the applied force. Furthermore, the relationship between these responses changed according to the coefficients of static friction. The experimental result indicated the proposed sensor could determine the coefficient of static friction before a global slip occurs.

## 1. Introduction

A tactile sensor is essential for dexterous manipulation of robot hands [1]. In real environments, the parameters regarding manipulation of an object, such as the coefficient of static friction, are unknown; as a result, performing grasping tasks is difficult. The coefficient of static friction is the threshold of the ratio of the static frictional force to the normal force when a slip occurs [2]. If the coefficient of static friction is clarified, then incident slips between the robot hand and the object surface can be prevented by controlling optimal force. Therefore, sensing the coefficient of static friction plays a significant role in the operation of dexterous robot hands. However, the coefficient of static friction is generally unknown before a slip actually occurs.

It is known that a slip starts in localized regions; such a slip is defined as a local slip [3]. Subsequently, the local slip propagates to the surrounding contact regions and eventually causes a global slip [4,5,6]. A local slip occurs from regions where the ratio of the shear stress to the normal stress exceeds the coefficient of static friction. Human skin can sense coefficient of static friction by detecting local slips on the contact area between a fingertip and an object surface [7,8]. Modeling this ability of human skin, some researchers have attempted to achieve tactile sensors for detection of coefficient of static friction [9,10]. In these studies, an acoustic transmitter and receiver measuring the resonant frequency of a cavity in a silicone rubber [9] or strain gauges embedded in a soft robot finger [10] were used to detect local slips. Local slips were detected as strain changes of the elastomer embedding the sensor elements. These methods demonstrated that strain changes could clarify local slips and result in estimations of the coefficient of static friction between the sensors and an object. However, further miniaturization is required for these methods to be used as a tactile sensor.

Alternatively, several types of MEMS tactile sensors have been reported [11]. MEMS tactile sensors have several advantages for actual use, for example, miniaturization or multiple functions. Recently, high performance tactile sensors are able to measure not only normal force but also shear force [12,13,14,15]. We have also been developing MEMS multi-axial tactile sensors using flat piezoresistive beams [16,17]. By forming piezoresistors on the surface of a pair of beams, a sensing element that is sensitive to only the vertical deformation of the beams can be achieved. Moreover, by forming piezoresistors on the sidewall of a pair of beams, a sensing element that is sensitive to only the lateral deformation of the beams can be achieved. The crosstalk between the normal force and the shear force is small because of these characteristics. The sensor is also easily miniaturized due to its simple structure. Thus, the developed sensing element is suitable as a tactile sensor. Providing that the function of sensing coefficient of static friction is added to the previous sensor, MEMS sensors will be more beneficial as a tactile sensor.

We propose a tactile sensor using piezoresistive beams for detection of the coefficient of static friction. In the proposed sensor, three pairs of surface-doped or sidewall-doped piezoresistive beams are arranged in parallel. The coefficient of static friction is estimated from the difference of the fractional resistance changes of the piezoresistors when normal force is applied on the sensor surface.

The estimation of coefficient of static friction is robust against the shear force; the effect of shear stress to the estimation of coefficient of static friction is cancelled by combining the outputs from two pairs of sidewall-doped beams, whose responses to shear force are similar to each other. Furthermore, the proposed structure is compatible with fabrication processes of the multi-axial tactile sensor using piezoresistive beams reported previously [16,17]. Therefore, by combining both sensors, simultaneous detections of multi-axial force and the coefficient of static friction will be achieved. The simultaneous detections of forces and the coefficient of static friction are significant issue to control robot hands with an optimal force to prevent a slip.

In this paper, we introduce the design, FEM simulation and fabrication process of the sensor chip, which was embedded in PDMS. Next, the fabricated sensor was evaluated under conditions of a range of coefficients of static friction.

## 2. Principle

**Figure 1:**
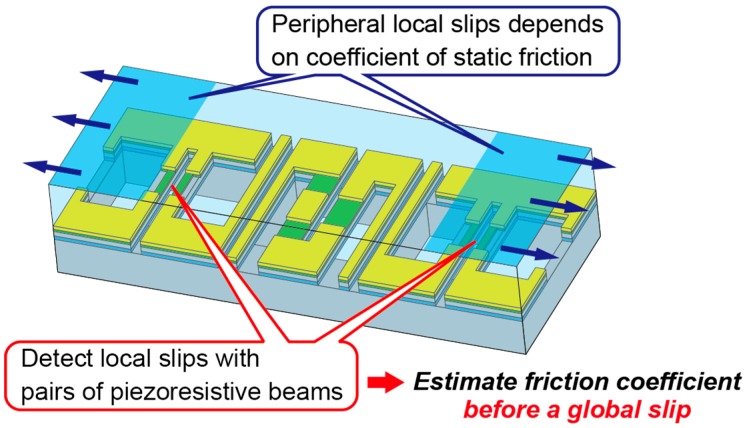
Conceptual sketch of the proposed sensor.

Figure 1 shows the conceptual sketch of the proposed sensor. The proposed sensor is composed of an elastomer and a sensor chip embedded in it. The sensor chip is composed of three pairs of silicon beams with piezoresistors. Because of the differences of the position of piezoresistors, the central pair of surface-doped silicon beams detects the vertical deformation, and the other two pairs of sidewall-doped silicon beams detect the lateral deformations. The deformations of the beams are small enough that the independency between the vertical and lateral deformation is satisfied.

The vertical and lateral strains of the elastomer are caused by a normal force compressing it. Here, the vertical strain is defined as the contraction in the same direction as the normal force; the lateral strain is defined as the horizontal expansion. Therefore, the vertical and lateral strains of the elastomer can be independently measured by the surface-doped beams and the sidewall-doped beams, respectively. The vertical strain of the elastomer mainly depends on the normal force applied on the sensor surface, whereas the lateral strain is mainly influenced by the proportion of local slip when normal force is applied because local slip causes the lateral deformation of the elastomer. Moreover, the proportion of the local slip depends on coefficient of static friction. Hence, the vertical and lateral strains of the elastomer correlate with the normal force and the coefficient of static friction, respectively. The lateral strain also depends on the normal force. Thus, the coefficient of static friction can be estimated from the lateral strain and the vertical strain.

In addition, a shear force uniformly applied on the sensor surface has no effect to the estimation. The uniform shear deformation of the elastomer at the surface is independent of the vertical and horizontal strains caused by the normal force because they are perpendicular. We experimentally observed that the effect was small enough to be ignored, as shown in Section 4.4.

**Figure 2:**
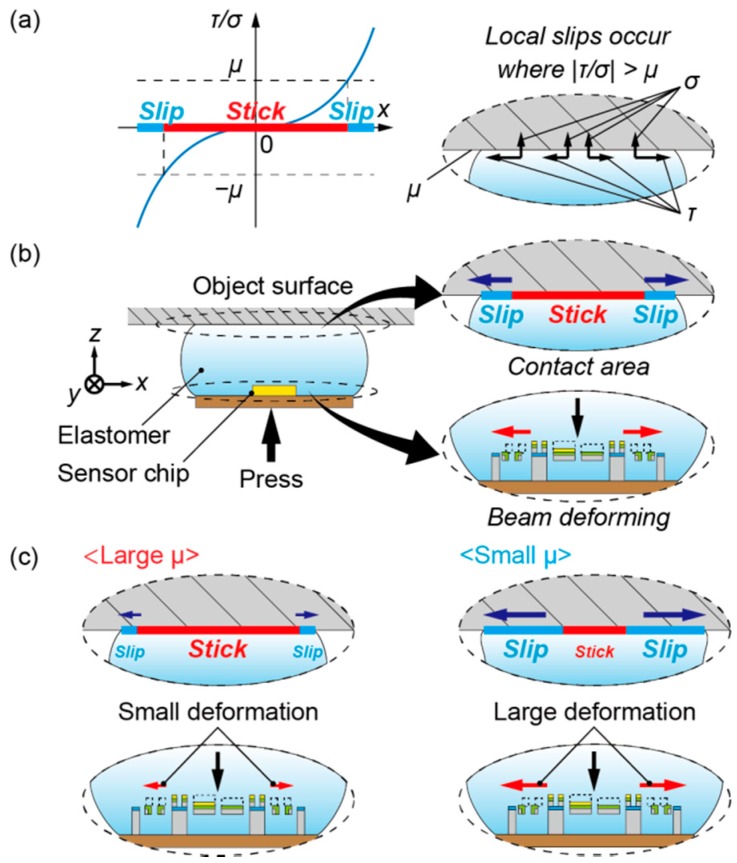
Measurement principle of the proposed sensor. (**a**) Mechanism of local slip on the contact area, where *σ* is the normal stress, *τ* is the shear stress and μ is the coefficient of static friction; (**b**) Situation where the sensor is pressed against an object surface; (**c**) Difference of deformations of the beams between large and small friction coefficients.

Figure 2 shows the measurement principle of the proposed sensor. Figure 2a shows the mechanism of local slips. When an elastomer is vertically pressed against a rigid surface, normal stress and shear stress occur on the contact area because the elastomer tends to deform against normal reaction and frictional forces. The graph in Figure 2a shows the stress distribution when no slip occurs on the contact area. In the region where the ratio of shear stress to normal stress exceeds the coefficient of static friction, a local slip will occur; we define this region as the slip area. In contrast, in the region where the ratio of shear stress to normal stress is less than the coefficient of static friction, no slip will occur; we define this region as the stick area. Assuming that the ratio of shear stress to normal stress becomes gradually larger from the center, the outside area can be regarded as the slip area and the center area can be regarded as the stick area. Under this assumption, the threshold points between the slip and stick area are determined by the stress distribution and the coefficient of static friction; the point where the ratio of stresses *τ/σ* becomes larger than the coefficient of static friction µ. We conduct the simulations and confirmed that this assumption is correct, as shown in Section 3.2.

The situation where the proposed sensor is pressed against an object surface is shown in Figure 2b. In this situation, normal and shear stress distributions exist on the contact area, and the slip area exists on the outside whereas the stick area exists on the center, as mentioned above. Alternatively, considering the sensor chip embedded in the elastomer, the beam structure deforms following the deformation of the elastomer. The deformations of the piezoresistive beams can be measured as electric signals using the same method as in a previous report [16]. The beam deformations differ depending on whether the coefficient of static friction is large or small, as shown in Figure 2c. In principle, local slip causes the spread of the contact area. Therefore, the deformation of the elastomer varies according to the proportion of the local slip. The proportion of the local slip is determined by the coefficient of static friction, as mentioned above. Thus, the deformation of each beam also depends on the friction coefficient. On one hand, under a large coefficient of static friction, the slip area is relatively small, and the lateral deformation of the sidewall-doped beams becomes small. On the other hand, under a small coefficient of static friction, the slip area is relatively large, and the lateral deformation of the sidewall-doped beams becomes large. Hence, the difference of the friction coefficient can be detected as the difference between the deformations of the piezoresistive beams.

## 3. Design and Fabrication

### 3.1. Sensor Design

**Figure 3:**
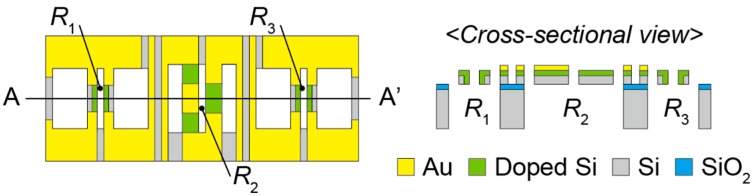
Design of the sensor chip.

Figure 3 shows the design of the sensor chip. We define three pairs of piezoresistive beams as *R*_1_, *R*_2_ and *R*_3_. These beams are arranged in parallel and fabricated in a 2 mm × 2 mm × 0.3 mm SOI wafer. The length and width of beams of *R*_1_ or *R*_3_ are 180 μm and 15 μm, respectively. The length and width of beams of *R*_2_ are 250 μm and 50 μm, respectively. The thickness of each beam is 20 μm. The gap of two beams of *R*_1_ or *R*_3_ is 35 μm. The gap of two beams of *R*_2_ is 30 μm. The distance between *R*_1_ and *R*_2_ is 600 μm. The distance between *R*_2_ and *R*_3_ is also 600 μm. A gold layer is formed as the wiring layer or electrode on a device silicon layer. The edge of each beam of *R*_1_ or *R*_3_ is covered with the gold layer that can be as long as 40 μm. One of two beams of *R*_2_ is covered by a gold layer of up to 65 μm from each edge; the center of the other beam of *R*_2_ is covered by a gold layer as long as 120 μm. The dimensions of through hole below *R*_1_ or *R*_3_ are 180 μm × 400 μm. The dimensions of the through hole below *R*_2_ are 250 μm × 250 μm.

### 3.2. FEM Simulation

**Figure 4:**
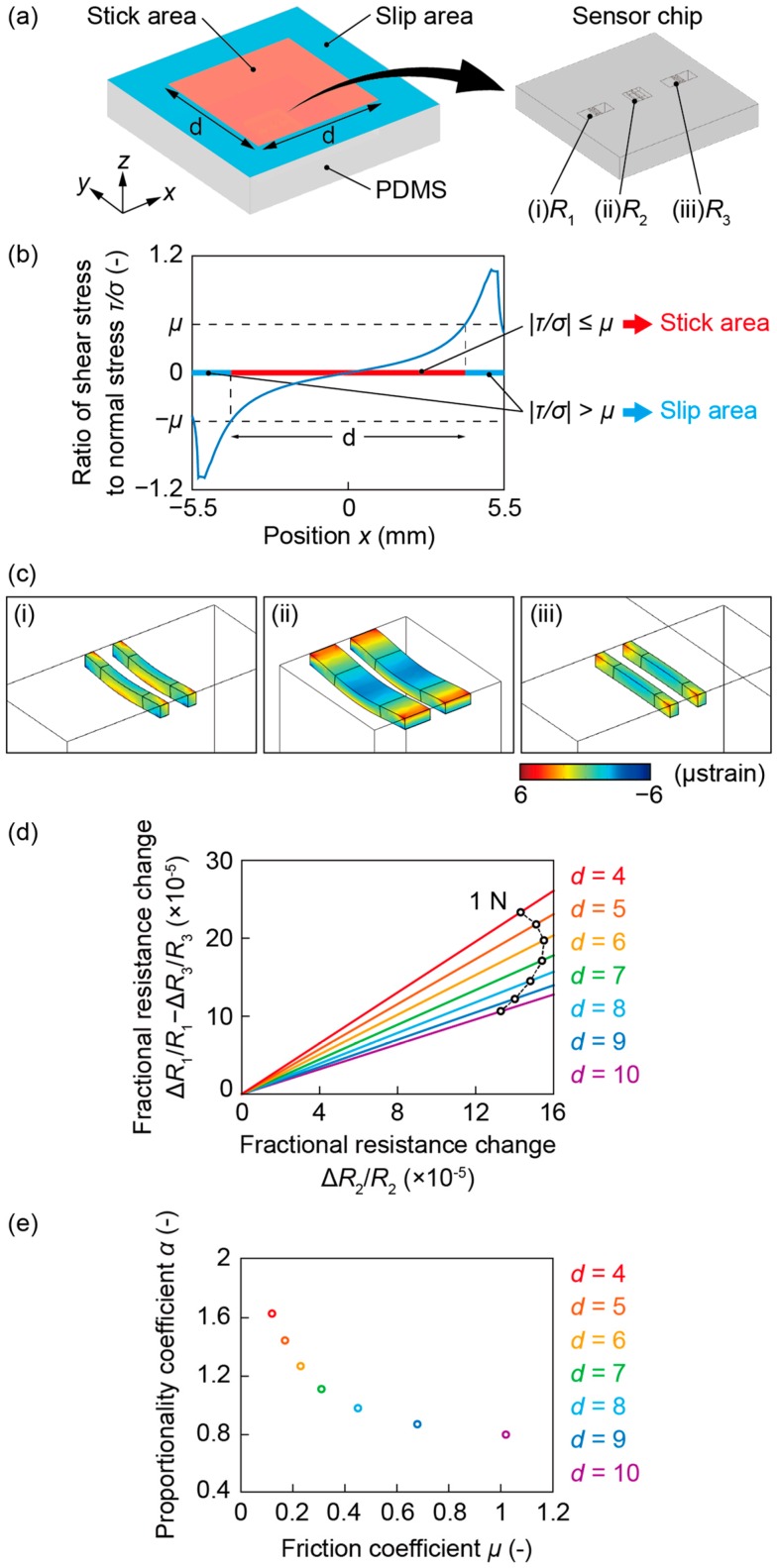
Simulation results. (**a**) The developed model; (**b**) Stress distribution on the contact area; (**c**) Deformation of the beams; (**d**) Relationship between two fractional resistance changes, Δ*R*_2_/*R*_2_ and Δ*R*_1_/*R*_1_—Δ*R*_3_/*R*_3_; (**e**) Relationship between the proportional coefficient *α* and the friction coefficient *μ*.

Figure 4 shows the simulation results using FEM software (COMSOL Multiphysics, COMSOL, Burlington, MA, USA). Figure 4a shows the developed model, which was composed of a sensor chip made of silicon and PDMS. The Young’s moduli of silicon and PDMS were 170 GPa and 600 kPa, respectively. The dimensions of the PDMS were defined as 11 mm × 11 mm × 2 mm. The sensor chip was embedded in the PDMS and fixed on the bottom surface. The stick area was defined as a fixed membrane on the central *d* mm square area of the top surface. The slip area was defined as a free membrane on the periphery area. The normal displacement was applied on the PDMS surface for different values of *d*: 4, 5, 6, 7, 8, 9, 10 and 11 mm. The applied normal force was 1 N.

Figure 4b shows the stress distribution when *d* was 11 mm, *i.e.*, the contact area was completely stuck. This result verifies that the assumption that the ratio of shear stress to normal stress becomes gradually larger from the center toward the outside is correct. Furthermore, the result also shows that a local slip surely occurs at the edge of the elastomer regardless of the magnitude of applied normal force. It is because the ratio of shear stress to normal stress is independent of the magnitude of applied normal force. Therefore, *μ* can be estimated from the value of this graph at the point where *x* is *d*. For example, when *d* was 7 mm, *μ* was approximately 0.31.

Figure 4c shows the deformation of each pair of beams when *d* was 7 mm. *R*_1_ and *R*_3_ deformed toward the opposite direction against each other, as shown in Figure 4c (i) and Figure 4c (iii). The average strains where piezoresistors were formed of *R*_1_ and *R*_3_ were 8.5 × 10^−7^ and −8.5 × 10^−7^, respectively, and that of *R*_2_ was 1.5 × 10^−6^. Assuming that the gauge factor of piezoresistors on (1,0,0) crystal plane of n-doped silicon was 100, the fractional resistance changes of *R*_1_, *R*_2_ and *R*_3_ were 8.5 × 10^−5^, 1.5 × 10^−4^ and −8.5 × 10^−5^, respectively.

Figure 4d shows the relationship between two fractional resistance changes, Δ*R*_2_/*R*_2_ and Δ*R*_1_/*R*_1_—Δ*R*_3_/*R*_3_. The trajectories described by Δ*R*_2_/*R*_2_ and Δ*R*_1_/*R*_1_—Δ*R*_3_/*R*_3_ with the change of the applied normal force were linear. However, the proportionality coefficient *α* varied with *d*. For example, under 1 N of the normal force, Δ*R*_2_/*R*_2_ was 1.5 × 10^−4^ and Δ*R*_1_/*R*_1_—Δ*R*_3_/*R*_3_ was 1.7 × 10^−4^ when *d* was 7 mm; *α* is 1.1. Alternatively, Δ*R*_2_/*R*_2_ was 1.3 × 10^−4^ and Δ*R*_1_/*R*_1_—Δ*R*_3_/*R*_3_ was 1.1 × 10^−4^ when *d* was 10 mm; *α* is 0.8. Figure 4e shows the relationship between the friction coefficient *μ* estimated from Figure 4b and the proportional coefficient *α* of Figure 4d. The simulation result shows that *α* increases as *μ* decreases. Therefore, using this relationship, *μ* can be estimated from *α*, which is to be acquired from sensor signals.

### 3.3. Fabrication Process

**Figure 5:**
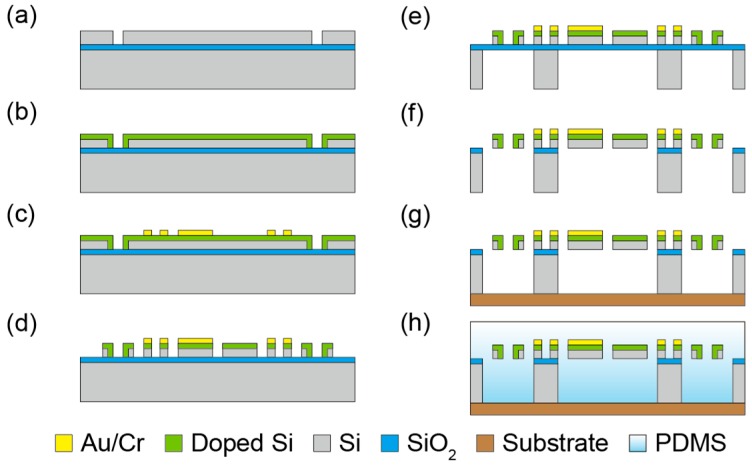
Fabrication process. (**a**) Etch device Si; (**b**) Dope P_2_O_5_ to the top and side walls of the device Si; (**c**) Form Au/Cr wirings to the device Si layer with lift off method; (**d**) Pattern device Si to form beams; (**e**) Remove handle Si; (**f**) Remove SiO_2_ to release beams; (**g**) Attach fabricated sensor element to flexible wiring substrate; (**h**) Embed whole structure to PDMS.

Figure 5 shows the fabrication process of the sensor chip. A 20/1/300 μm SOI wafer was used as the starting material. In this process, the silicon was etched by using ICP-RIE. First, holes for sidewall-doping were etched on the device silicon layer, as shown in Figure 5a. Second, phosphorus ions were doped on the device silicon layer by thermal diffusion method [18,19,20], as shown in Figure 5b. Third, a gold/chrome layer was patterned on the doped layer by a liftoff process, as shown in Figure 5c. Fourth, the beam structure was formed by etching the device silicon layer again, as shown in Figure 5d. Fifth, the handle silicon layer was etched from the backside, as shown in Figure 5e. Last, the silicon dioxide layer was etched with hydrofluoric acid vapor, and then the beam structure was released, as shown in Figure 5f. The fabricated sensor chip was fixed with an epoxy adhesive on the substrate, electrically connected by conductive paste (Dotite D-753, Fujikura Kasei, Tokyo, Japan), and then embedded in 11 mm × 11 mm × 2 mm PDMS, as shown in Figure 5g, h. The mixture ratio of the PDMS and its curing agent was 10:1. The PDMS was cured on a horizontal table to obtain the surface flatness and the thickness uniformity over the sensor chip.

**Figure 6:**
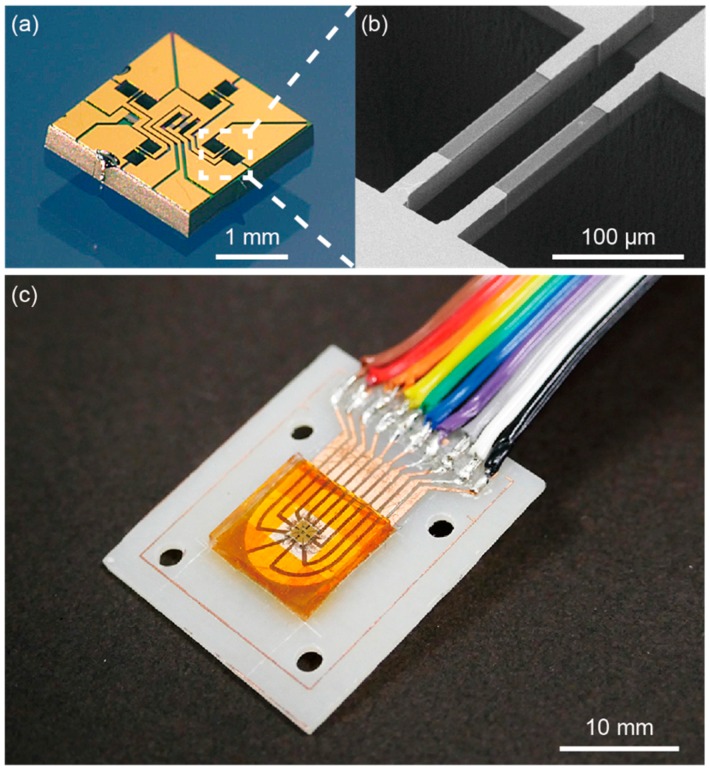
(**a**) Photograph of the sensor chip; (**b**) SEM image of the beams; (**c**) Photograph of the prototype of the proposed sensor.

Figure 6a–c show photographs of the fabricated sensor chip, the SEM image of a pair of sidewall-doped beams and the prototype of the proposed sensor, respectively.

## 4. Experiment and Result

### 4.1. Setup and Trial

**Figure 7:**
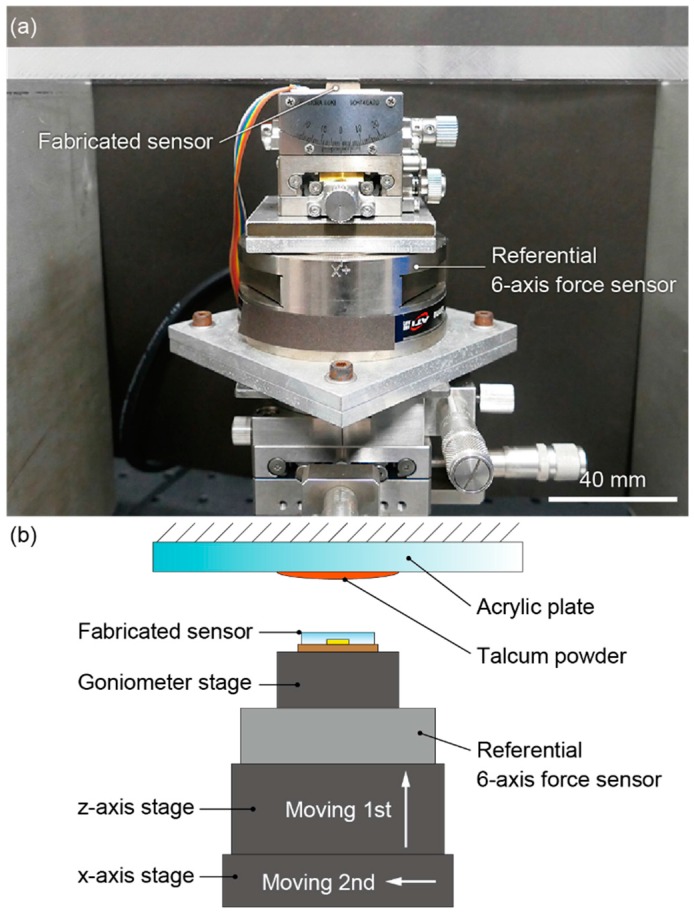
(**a**) Photograph and (**b**) schematic image of the experimental setup.

Figure 7a,b shows the photograph and the schematic image of the experimental setup, respectively. The fabricated sensor was fixed on the referential 6-axis force sensor (SI-130-10, ATI Industrial Automation, Apex, NC, USA) together with an XYZ-axes manual stage and a goniometer stage. An acrylic plate was fixed above the fabricated sensor. The fractional resistance changes of pairs of piezoresistive beams were measured using a Wheatstone bridge circuit. The signals were amplified 250-fold by instrumentation amplifiers (AD623, Analog Devices, Tokyo, Japan) and recorded by an oscilloscope. The source voltage of the bridge circuit was 1 V.

A trial was conducted as follows. First, talcum powder (Baby Powder, Johnson & Johnson, New Brunswick, NJ, USA) was coated on the acrylic plate by wiping it with a cloth. The amount of talcum powder was changed for each trial so that the coefficient of static friction ranged from 0.2 to 1.1. Next, by moving the Z-axis stage, the normal force was applied to the fabricated sensor by pressing it against the acrylic plate, as described in Figure 7b. Last, the shear force was applied by moving the X-axis stage in parallel to the acrylic plate. A global slip occurred when the stage moved by a certain extent. Accordingly, the friction coefficient in this trial was acquired as the ratio of the normal force to the shear force measured by the 6-axis force sensor when the first global slip occurred. After this trial, the acrylic plate was cleaned, and the next trial was conducted. In this experiment, 14 trials were conducted in total.

### 4.2. Response to Normal Force

**Figure 8:**
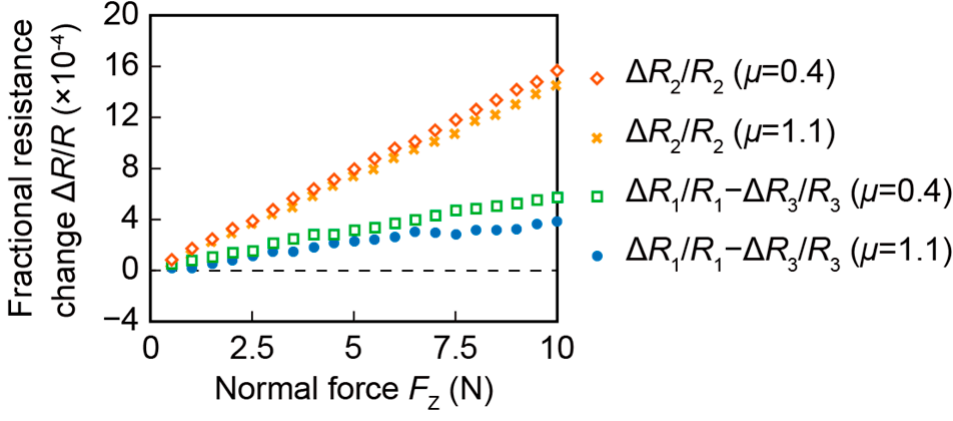
Response of the fabricated sensor to the normal force.

Figure 8 shows the fractional resistance changes, Δ*R*_2_/*R*_2_ and Δ*R*_1_/*R*_1_—Δ*R*_3_/*R*_3_, to the normal force *F*_Z_ when the coefficient of static friction *μ* was 0.4 and 1.1. Both Δ*R*_2_/*R*_2_ and Δ*R*_1_/*R*_1_—Δ*R*_3_/*R*_3_ were proportional to the normal force, independent of the differences of coefficients of static friction. The sensitivity was defined as the ratio of fractional resistance change to the normal force. On one hand, the sensitivities of Δ*R*_2_/*R*_2_ were 1.5 × 10^−4^ *F*_Z_ and 1.6 × 10^−4^ *F*_Z_ when *μ* were 0.4 and 1.1, respectively (units for *F*_Z_ is N). On the other hand, the sensitivities of Δ*R*_1_/*R*_1_—Δ*R*_3_/*R*_3_ were 6.1 × 10^−5^ *F*_Z_ and 4.2 × 10^−5^ *F*_Z_ when *μ* were 0.4 and 1.1, respectively. This result appears to match the simulation result that the deformations of the beams vary according to the coefficient of static friction. Moreover, the sensitivity of Δ*R*_2_/*R*_2_ was approximately equal to that acquired in the simulation. However, the sensitivity of Δ*R*_1_/*R*_1_—Δ*R*_3_/*R*_3_ is equal to half of the simulation result. The reason for this is thought to be that the sidewall-doped beams had extra parallel piezoresistors on the surface, as mentioned in the previous report [16].

### 4.3. Response to Friction Coefficient

**Figure 9:**
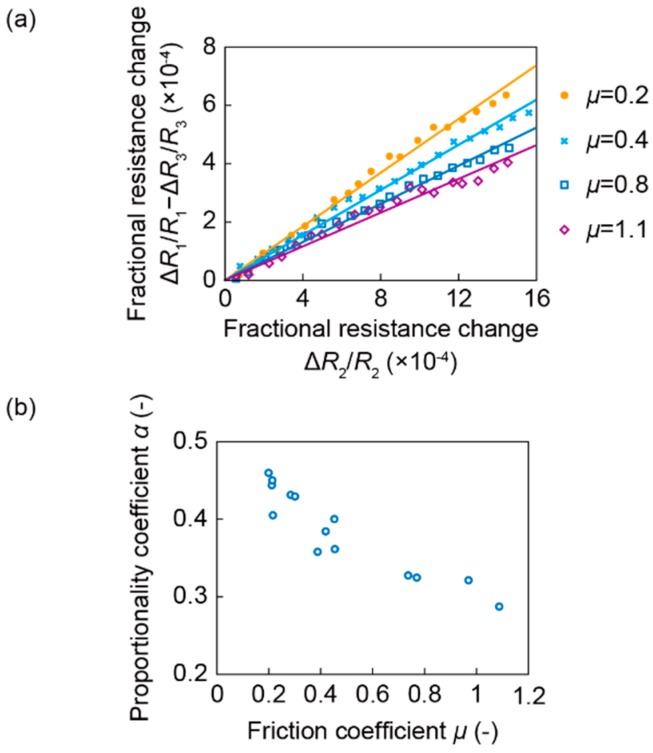
(**a**) Relationship between two fractional resistance changes, Δ*R*_2_/*R*_2_ and Δ*R*_1_/*R*_1_—Δ*R*_3_/*R*_3_; (**b**) Relationship between the proportional coefficient *α* and the friction coefficient *μ*.

Figure 9a shows the relationship between Δ*R*_2_/*R*_2_ and Δ*R*_1_/*R*_1_—Δ*R*_3_/*R*_3_ when *μ* is 0.2, 0.4, 0.8 and 1.1. The trajectories described by Δ*R*_2_/*R*_2_ and Δ*R*_1_/*R*_1_—Δ*R*_3_/*R*_3_ were linear. Furthermore, the proportionality coefficient *α* of the fitting line varied with *μ*: *α* became larger when *μ* became smaller. For example, *α* were 0.39 and 0.29 when *μ* were 0.4 and 1.1, respectively. This result corresponded with the simulation result, and verified that the sensor output showed the signals after a local slip that surely occurred at the edge of the elastomer.

Figure 9b shows the relationship between *μ* obtained in each trial and *α* plotted for all of 14 trials. It was confirmed that *α* increased while *μ* decreased, in agreement with the simulation result. Therefore, using this relationship, *μ* was able to be estimated from *α*. The value of *α* calculated in the experiment was approximately half of that of the simulation because the sensitivity of Δ*R*_1_/*R*_1_ – Δ*R*_3_/*R*_3_ is half of that of the simulation, as mentioned above.

In actual use, the experimentally-obtained relationship can be applied to estimate the coefficient of static friction by calibrating the sensor and obtaining the characteristics such as estimation accuracy.

We proposed a tactile sensing unit embedded in a piece of elastomer. In actual use, the piece must be isolated to other pieces because otherwise the lateral deformation characteristics of the elastomer against the local slippage will change.

### 4.4. Response to Shear Force

**Figure 10:**
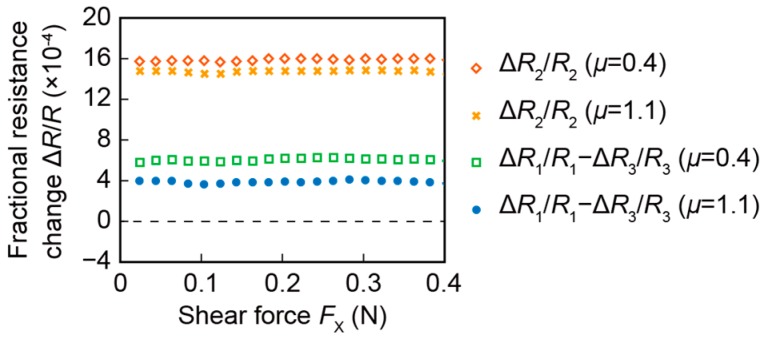
Response of the fabricated sensor to the shear force.

Figure 10 shows the fractional resistance changes, Δ*R*_2_/*R*_2_ and Δ*R*_1_/*R*_1_—Δ*R*_3_/*R*_3_, to the shear force *F*_X_ with the coefficient of static friction *μ* of 0.4 or 1.1. After applying a normal force, the shear force was gradually applied. Both Δ*R*_2_/*R*_2_ and Δ*R*_1_/*R*_1_—Δ*R*_3_/*R*_3_ were kept constant against the shear force; these values did not change from the initial values with the normal force. In actual use, some unintentional shear force is possibly applied on the sensor surface. As a result, the robustness against shear force is required for the estimation of the coefficient of static friction. The experimental result shows that the proposed sensor was robust against shear force.

## 5. Conclusions

In this paper, we proposed a tactile sensor using piezoresistive beams embedded in an elastomer for detection of the coefficient of static friction. The size of the fabricated sensor was 11 mm × 11 mm × 2 mm, and it was evaluated under various friction coefficients from 0.2 to 1.1. The fractional resistance changes corresponding to vertical and lateral strains of the elastomer were proportional to the applied normal force. It was confirmed that the proportional coefficient of the relationship between these responses varied according to coefficient of static friction. Thus, the characteristics lead to the estimation of the coefficient of static friction before a global slip occurs. A tactile sensor fabricated by the proposed method would be useful for robotics applications.

## Abbreviations

The following abbreviations are used in this manuscript:

MEMS	Micro Electro Mechanical Systems
FEM	Finite element method
ICP-RIE	Inductive Coupled Plasma-Reactive Ion Etching
PDMS	Polydimethylsiloxane
SOI	Silicon-on-insulator
SEM	Scanning Electron Microscope

## References

[1] Kappassov Z., Corrales J.-A., Perdereau V. (2015). Tactile sensing in dexterous robot hands—Review. Robot. Auton. Syst..

[2] Persson B.N.J. (1998). Sliding Friction.

[3] Johnson K.L. (1985). Contact Mechanics.

[4] Maeno T., Hiromitsu S., Kawai T. Control of grasping force by detecting stick/slip distribution at the curved surface of an elastic finger. Proceedings of the IEEE International Conference on Robotics and Automation (ICRA 2000).

[5] Nguyen T.-V., Takahashi H., Nguyen B.-K., Matsumoto K., Shimoyama I. Measurement of the pressure distribution during the onset of slip. Proceedings of the IEEE Sensors 2012.

[6] Ho V.-A., Hirai S. (2011). Modeling and analysis of a frictional sliding soft fingertip, and experimental validations. Adv. Robot..

[7] Johansson R.S., Flanagan J.R. (2009). Coding and use of tactile signals from the fingertips in object manipulation tasks. Nat. Neurosci..

[8] Ho V.-A., Hirai S. (2015). A novel model for assessing sliding mechanics and tactile sensation of human-like fingertips during slip action. Robot. Auton. Syst..

[9] Nakamura K., Shinoda H. A tactile sensor instantaneously evaluating friction coefficients. Proceedings of the 11th International Conference on Solid-State Sensors, Actuators and Microsystems.

[10] Maeno T., Kawamura T., Cheng S.C. (2004). Friction estimation by pressing an elastic finger-shaped sensor against a surface. IEEE Trans. Robot. Autom..

[11] Yousef H., Boukallel M., Althoefer K. (2011). Tactile sensing for dexterous in-hand manipulation in robotics—A review. Sens. Actuators A Phys..

[12] Noda K., Hoshino K., Matsumoto K., Shimoyama I. (2006). A shear stress sensor for tactile sensing with the piezoresistive cantilever standing in elastic material. Sens. Actuators A Phys..

[13] Charalambides A., Cheng J., Li T., Bergbreiter S. 3-axis all elastomer MEMS tactile sensor. Proceedings of the 28th IEEE International Conference on Micro Electro Mechanical Systems (MEMS 2015).

[14] Xi K., Wang Y., Mei D., Liang G., Chen Z. A flexible sensor array based on pressure conductive rubber for three-axis force and slip detection. Proceedings of the IEEE International Conference on Advanced Intelligent Mechatronics (AIM 2015).

[15] Asano S., Muroyama M., Bartley T., Nakayama T., Yamaguchi U., Yamada H., Hata Y., Nonomura Y., Tanaka S. 3-axis fully-integrated surface-mountable differential capacitive sensor by CMOS flip-bonding. Proceedings of the 29th IEEE International Conference on Micro Electro Mechanical Systems (MEMS 2016).

[16] Takahashi H., Nakai A., Nguyen T.-V., Matsumoto K., Shimoyama I. (2013). A triaxial tactile sensor without crosstalk using pairs of piezoresistive beams with sidewall doping. Sens. Actuators A Phys..

[17] Nakai A., Morishita Y., Matsumoto K., Shimoyama I. 6-axis force-torque sensor chip composed of 16 piezoresistive beams. Proceedings of the 28th IEEE International Conference on Micro Electro Mechanical Systems (MEMS 2015).

[18] Kan T., Takahashi H., Nguyen B.-K., Aoyama Y., Takei Y., Noda K., Matsumoto K., Shimoyama I. (2013). Design of a piezoresistive triaxial force sensor probe using the sidewall doping method. J. Micromech. Microeng..

[19] Gel M., Shimoyama I. (2004). Force sensing sub micrometer thick cantilevers with ultra-thin piezoresistors by rapid thermal diffusion. J. Micromech. Microeng..

[20] Isozaki A., Kuwana K., Tomimatsu T., Itoh T. Photodiode with micro texture for improving sensitivity at large angle of incidence for particle sensors. Proceedings of the 16th International Conference on Solid-State Sensors, Actuators and Microsystems (TRANSDUCERS 2011).

